# Role of Metformin on Osteoblast Differentiation in Type 2 Diabetes

**DOI:** 10.1155/2019/9203934

**Published:** 2019-11-26

**Authors:** Lin Jiating, Ji Buyun, Zhang Yinchang

**Affiliations:** ^1^Department of Stomatology, The First Affiliated Hospital of Wannan Medical College, Wuhu, Anhui Province 241000, China; ^2^Department of Orthopedics, The First Affiliated Hospital of Wannan Medical College, Wuhu, Anhui Province 241000, China

## Abstract

Metformin, an effective hypoglycemic, can modulate different points of malignant mass, polycystic ovary syndrome (PCOS), cardiovascular diseases, tuberculosis, and nerve regeneration. Recently, the effect of metformin on bone metabolism has been analyzed. Metformin relies on organic cation transporters (OCT1), a polyspecific cell membrane of the solute carrier 22A (SLC22A) gene family, to facilitate its intracellular uptake and action on complex I of the respiratory chain of mitochondria. These changes activate the cellular energy sensor AMP-activated protein kinase (AMPK). Thus, the increased cellular AMP/ATP ratio causes a dramatic and progressive activation of insulin and lysosomes, resulting in a decrease in intracellular glucose level, which promotes osteoblast proliferation and differentiation. AMPK also phosphorylates runt-related transcription factor 2 (Runx2) at S118, the lineage-specific transcriptional regulators, to promote osteogenesis. Metformin phosphorylates extracellular signal-regulated kinase (ERK), stimulates endothelial and inducible nitric oxide synthases (e/iNOS), inhibits the GSK3*β*/Wnt/*β*-catenin pathway, and promotes osteogenic differentiation of osteoblasts. The effect of metformin on hyperglycemia decreases intracellular reactive oxygen species (ROS) and advanced glycation end-products (AGEs) in collagen, and reduced serum levels of insulin-like growth factors (IGF-1) were beneficial for bone formation. Metformin has a certain effect on microangiopathy and anti-inflammation, which can induce osteoporosis, activate the activity of osteoclasts, and inhibit osteoblast activity, and has demonstrated extensive alteration in bone and mineral metabolism. The aim of this review was to elucidate the mechanisms of metformin on osteoblasts in insulin-deficient diabetes.

## 1. Introduction

Metformin, a wide spectrum of efficacy, safety hypoglycemic agents [1, 2], was introduced as a medication for type 2 diabetes (T2D) in 1957. As the first-line oral antihyperglycemic agent, metformin is recommended in all stages of either monotherapy or the therapy combined with other oral antihyperglycemic drugs and insulin [1, 3, 4]. Metformin exerts its effect on insulin-sensitive organs or tissues, such as the liver, skeletal muscle, and adipose tissues [5]. It regulates glucose homeostasis mainly by inhibiting hepatic gluconeogenesis [6]. Data have showed the beneficial effects of metformin on diabetes-associated conditions, like malignancies [7–10], inflammation [11–13], and heart failure [14]. The effect of metformin on bone metabolism in humans has been studied. A multicenter study showed metformin treatment may decrease fasting bone turnover markers representing bone resorption, suggesting that metformin may have beneficial effects on bones in diabetic patients [15]. Metformin also decreased the fracture rate in patients in vivo [16, 17] and promoted the osteogenesis of osteoblasts in culture [18–20]. The findings showed that metformin could prevent bone loss in ovariectomized (OVX) rats and inhibit receptor activator of nuclear factor *k* B ligand- (RANKL-) induced osteoclast differentiation in Raw264.7 macrophage cells [21]. Moreover, a lot of data showed a long-term protective effect of metformin in bone metabolism [22, 23] of diabetic or prediabetic patients [24, 25].

Diabetes mellitus is manifested with abnormal bone mineral density (BMD), hyperglycemia, secondary calcium imbalance, disturbances in vitamin D [26], microvascular disease [27], and an increased risk of fall [28, 29]. With a high morbidity and mortality, it also adversely affects bone metabolism and increases the fracture risk [30–32]. Hyperglycemia also disrupts the production of ROS and AGE, which affects cell death processes [33, 34].

**Figure 1:**
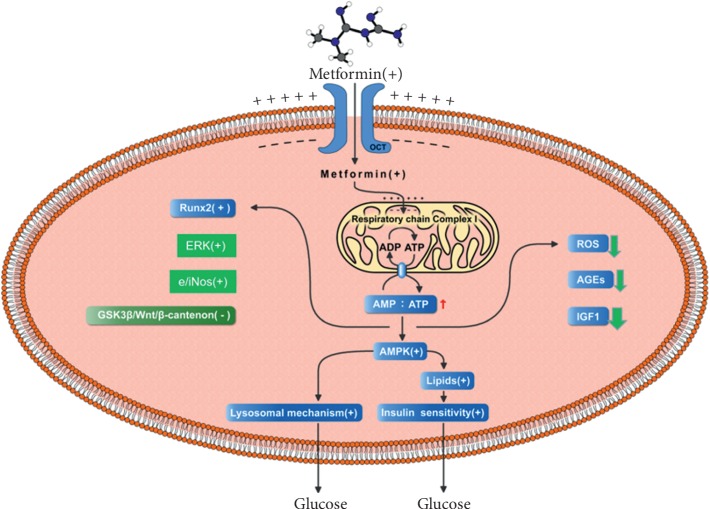
Potential mechanisms of metformin on osteoblasts in insulin-deficient diabetes. Metformin inhibits the respiratory chain complex I leading to an increase in AMP : ATP ratio and then stimulates 5ʹ-AMP-activated protein kinase (AMPK), promotes glycolipid metabolism, phosphorylates runt-related transcription factor 2 (Runx2) and extracellular signal-regulated kinase (ERK), stimulates endothelial and inducible nitric oxide synthases (e/iNOS), and reduces reactive oxygen species (ROS) production, advanced glycation end-products (AGEs), and insulin-like growth factors (IGF-1).

Several studies have reported that metformin has a potential osteogenic effect by promoting the differentiation of preosteoblasts and MSCs [19, 35, 36]. Likewise, metformin can also revert the deleterious effects of high-level glucose on osteoblastic cells [37–39]. Clinical studies have also indicated that locally delivered metformin can normalize clinical parameters of chronic periodontitis [40]. How metformin affects osteoblast differentiation is undetermined. In the present review, we explored the molecular mechanisms of metformin on osteoblasts in type 2 diabetes (Figure 1).

## 2. Methods

Using the following terms, “bone,” “skeleton,” “osteoblasts,” “osteocytes,” “osteoclasts,” “bone abnormalities,” “osteoporosis,” “osteopenia,” “idiopathic skeletal hyperostosis,” “perimenopausal bone loss,” “bone metabolism,” “insulin sensitivity,” “type 2 diabetes,” “prediabetes,” “metformin,” and “biguanides” were searched in PubMed and the Cochrane Library for RCTs, reviews, and meta-analyses. We included relevant references in the aforementioned articles and performed hand searches for older publications.

## 3. Metformin and Cell Membrane Organic Cation Transporters (OCTs)

Metformin is a hydrophilic compound charged positively at physiological pH. Its hydrophilicity decides its permeability through lipid membranes. The hydrophility of metformin is achieved by the polyspecific cell membrane organic cation transporters (OCTs), the solute carrier 22A (SLC22A) gene family [41–43], and multidrug and toxin extrusion (MATEs), encoded by the gene SLC47A1 [42, 44, 45]. The transporters weaken bases in an electrogenic manner to facilitate the intracellular uptake and action of metformin [46]. The present study shows that osteoblasts can transport metformin into cells through active rOCT1 (SLC22A1), and also high-level glucose can improve the uptake of metformin by osteoblasts through phosphorylating rOCT1 [47], which leads to a intracellular concentration of metformin that is stable and associated with the amount and activity of organic cation transporters, as well as metformin plasma concentration.

Since metformin carries a positive charge and can cross the plasma membrane and mitochondrial inner membrane (positive outside), metformin can enter the mitochondria [48, 49], where the concentration of metformin can rise up to 1000-fold higher than that in the extracellular circumstance to provide sufficient energy for the following biochemical reaction.

## 4. Direct Effect of Metformin on Osteogenesis

### 4.1. AMP-Activated Protein Kinase (AMPK) Signaling Pathway

Recently, the action of metformin in mitochondria has been intensively studied by researchers aiming to find out the osteogenic mechanism of metformin. The inhibition of complex I of the respiratory chain was highly valued [48, 50, 51], for it was proven to suppress ATP production and increase the ratios of cytoplasmic ADP to ATP and AMP to ATP ratios (a drop in ATP level and an increase in AMP level). These changes activated AMP-activated protein kinase (AMPK) [52]. Thus, the increased cellular AMP/ATP ratio caused a progressive activation of AMPK.

Mammalian AMPK comprises *α*, *β*, and *γ* subunits in a heterotrimeric complex [53, 54]. The *α* subunit has two isoforms, *α*1 and *α*2, and contains a kinase domain at the N terminus that is phosphorylated at Thr172 by upstream kinases [55]. The *α*2 subunit is highly expressed in skeletal and cardiac muscles and the liver [56]. The AMPK subunits displayed differential tissue-specific expression and activation [57]. AMPK *α*2 subunit was highly expressed in bone tissues, primary osteoblasts, osteoclasts, and osteoblastic cell lines [58]. AMPK also limited energy utilization to ensure cell survival, so activation of AMPK can mediate cell cycle arrest, inhibit cell growth, and suppress protein synthesis through downregulating the mechanistic target of rapamycin (MTOR, also known as the mammalian target of rapamycin) signaling [59, 60]. Metformin has been uniformly shown as a potent stimulator of AMPK activation in osteoblasts [21, 61]. The downstream target of AMPK was also phosphorylated by metformin in primary osteoblasts and the acetyl-coA carboxylase 1 (ACC1) and ACC2 isoforms of ACC phosphorylated, a process inhibiting fat synthesis, promoting fat oxidation, enhancing insulin sensitivity [62], and consequently initiating the acute inhibition of gluconeogenesis [63].

Several studies have examined the effects of AMPK activators on osteoblast differentiation, showing that metformin harbored direct osteogenic effects, such as maintaining BMD, antagonizing bone loss, and protecting bone microarchitecture [15, 18, 24, 64]. AMPK signaling activation may stimulate bone formation and increase bone mass in skeletal physiology. However, the phosphorylation level of AMPK *α* subunits showed different results in the osteoblast differentiation, which was associated with decreased AMPK activity [58]. It is found that AMPK activity decreased with time during osteoblast differentiation, due to the high energy requirements and therefore elevated ATP levels in mature osteoblasts during bone matrix production and mineralization. Osteoblast differentiation coincided with the changes in cellular metabolism and mitochondrial activity [65]. Thus, metformin might activate the AMPK signal pathway in bone cells, and the mechanism linking AMPK activation with osteoblast differentiation and bone mass formation remains unknown.

### 4.2. Expression of Runt-Related Transcription Factor 2 (Runx2)

Recently studies indicated that metformin reduced the bone loss in vivo, partly through increasing bone formation via the induction of osteoblast genes such as Runx2 and Lrp5 [66], reducing receptor activator of RANKL level, and stimulating osteoprotegerin (OPG) expression in osteoblasts [21].

Runt-related transcription factor 2 (Runx2), a member of the runt domain gene family, promoted the differentiation of committed mesenchymal cells or preosteoblasts into osteoblasts by regulating a range of factors, such as alkaline phosphatase (ALP), osteocalcin (OCN), and bone sialoprotein (BSP) [67, 68]. Runx2 phosphorylation is critical in osteogenic commitment, and loss of Runx2 may activate the adipogenic process, which shows direct relation of AMPK-mediated Runx2 phosphorylation at serine 118 [69]. Metformin induced an osteogenic effect in vivo and in vitro, possibly mediated by Runx2 and activation of AMPK [19, 38, 70]. Metformin partially inhibited the adipogenic action of BMPC and stimulated bone healing in a craniotomy defect model in both control and diabetic rats [71]. Metformin was also found to stimulate osteoblastic bone formation in MC3T3-E1 cells by regulating small heterodimer partner, an orphan nuclear receptor, that can interact with Runx2 [18]. Metformin increased ALP activity, collagen synthesis, osteocalcin production, and extracellular calcium deposition in bone marrow mesenchymal cells in vitro, possibly by upregulating the expression of Runx2 [18, 38, 64]. While controversies remain, it seems certain that some of the acute effects of metformin regulate osteoblasts by the AMPK-Runx2 signal pathway.

### 4.3. Other Molecular Signal Pathways

Metformin induced activation of phosphorylated extracellular signal-regulated kinase (ERK) 1/2 (p42 and p44 MAPK) and stimulated the expression of endothelial and inducible nitric oxide synthases (e/iNOS) [19]. It has been showed that increased proliferation and differentiation of osteoblast-like cells (UMR106 and MC3T3E1) was accompanied with elevated type-I collagen production and ALP activity. Metformin promoted osteogenic differentiation of hBMSCs by inhibiting the GSK3*β*/Wnt/*β*-catenin pathway [20]. Moreover, metformin substantially increased mineralized nodule formation of iPSC-MSC by the LKB1/AMPK pathway [18].

## 5. Indirect Effect of Metformin on Osteogenesis

Diabetes affects bones through impairing glucose metabolism, disrupting bone microvascular function and muscle endocrine function, and producing glucose oxidative derivatives [72]. Chronic hyperglycemia contributed to bone loss by modulating osteoblast gene expression, function, and bone formation. Bone metabolism was disturbed by various abnormalities, including hyperinsulinemia, deposition of advanced glycosylation end-products (AGEs) in collagen, reduced serum levels of IGF-1, hypercalciuria, renal failure, microangiopathy, and inflammation [73]. Among them, AGEs impair osteoblastic function, increasing the risk of fracture and postpone fracture in type 2 diabetes [21–25].

High-level glucose (>44 mmol/L) significantly decreased the gene expression levels of Runx2, IGF-1, and IGF-1R [38], which inhibited osteoblastic proliferation and development. Metformin increased the expression of OPG and decreased the RANKL/OPG ratio in the implant medullary area, yielding some molecular benefits in the osseointegration of implants under the hyperglycemic state, which suppressed the cell growth and mineralization [74]. Moreover, high-level glucose increased the intracellular cAMP level in a time-dependent manner, which reduces bone mass and negatively regulates osteoclastogenesis [75]. These results indicate that high-level glucose can increase adipogenic and inhibit osteogenic differentiation in bone marrow stem cells.

Excessive accumulation of reactive oxygen species (ROS)—the radical forms of oxygen—contributes to age-related changes in bones [76, 77]. ROS affects not only osteogenesis but also stimulates osteoblastic apoptosis. Excessive generation of ROS failed bone regeneration in inflammatory conditions [78, 79]. The complications of diabetes accelerated osteoblasts aging and apoptosis although the exact mechanism is not distinctly.

Hyperglycemia, hyperinsulinemia, and excessive ROS alter the microenvironment of bone cells, disturb bone microstructure, decrease bone formation, increase bone resorption, and prolong fracture healing.

## 6. Conclusion and Future Perspectives

In conclusion, metformin, an activator of AMPK, may play an important role in osteoblast differentiation and bone formation. This may be associated with the direct effect of the AMPK signaling pathway, Runx2/AMPK signaling pathway, and the indirect effect or interaction effect of metformin. Recently, our experiment has found that local injection of metformin enhanced the autophagy of aging bone marrow stromal cells (BMSCs) in vivo and accelerated new bone formation of peri‐implant in vitro. The study, through the colony-forming efficiency assay, osteogenic induction and calcium accumulation, and analysis of oxidative stress, showed that metformin increased the stemness of jawbone BMSCs and accelerated new bone formation of peri‐implant, both contributing to the osseointegration. Our findings highlighted the therapeutic potential of metformin in site preservation and implant in stomatology. The exact intracellular mechanism of metformin on osteoblast is ominous, and it promotes osteogenesis unambiguously.
